# Donald Ainslie (D. A.) Henderson, MD, MPH (1928–2016) Smallpox Eradication: Leadership and Legacy

**DOI:** 10.1093/infdis/jiw640

**Published:** 2017-02-07

It was said in ancient Greece that when Aeschines spoke the crowd uttered, “What a great orator,” but when Demosthenes spoke they shouted, “Let us march!” Donald Ainslie (D. A. since childhood) Henderson became commanding general of the World Health Organization’s (WHO’s) Intensified Smallpox Eradication Programme in October 1966. To many of the more than 150000 pox-warriors who marched until the last case of naturally transmitted smallpox occurred in October 1977, he remains our revered leader (photo 1).

Henderson, who died at 87 on August 19, 2016, in Towson, Maryland, was born in Lakewood, Ohio, in 1928 to an engineer and a nurse. He went to Oberlin College in Ohio and trained in medicine at the University of Rochester in New York, where he wrote a prizewinning article about the 1833 epidemic of cholera in upstate New York. Initially considering cardiology as a specialty, he was a medical intern and resident at Mary Imogene Bassett Hospital in Cooperstown, New York.

In 1967, smallpox was endemic in 33 countries with a population of 1.2 billion, and importations occurred into 14 others. Disease surveillance was abysmal. Ten to 15 million cases a year were occurring, less than 5% ever being reported to health authorities, and up to one third of the patients would die and many others were scarred and blinded. There was no effective treatment—only traditional beliefs. Less than 10% of the smallpox vaccines in program use were of good quality. In 1959 the World Health Assembly in Geneva adopted a Soviet-inspired resolution for global smallpox eradication. However, little was accomplished—another resolution was passed at the assembly in 1966 allocating WHO funds to accelerate smallpox eradication; the resolution barely passed because of worry that a smallpox program could not succeed, would compete with the malaria eradication effort, and require more funding from member states.

**Photo 1.**
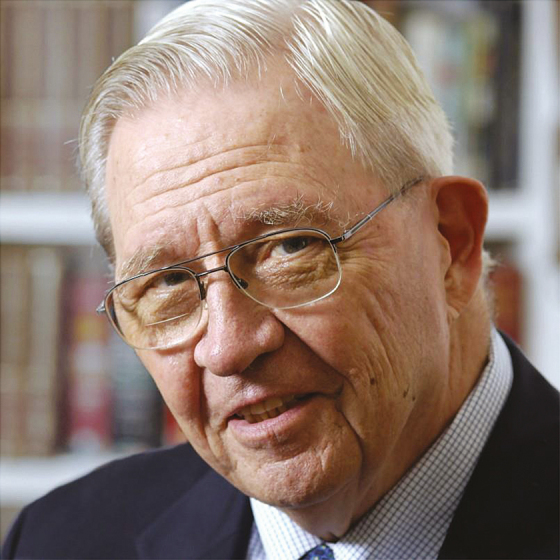
Donald Ainslie (D. A.) Henderson

Enter D. A. He came to WHO from the US Communicable Disease Center (CDC, now the Centers for Disease Control and Prevention). For 11 years, from 1955 to 1966, he was mentored by Alexander Langmuir, Epidemiology Branch chief and founder in 1951 of the famed Epidemic Intelligence Service (EIS), formed in part to detect and respond to biowarfare attacks in the United States following the Korean War. Langmuir was a demanding boss, emphasizing “shoe leather” investigations of disease outbreaks, close partnerships with state health departments, practical recommendations to solve public health problems, and precision in scientific reports. D. A.’s studies during this period targeted influenza, measles, polio, hepatitis, cholera, antibiotic resistance, epidemic neuromyasthenia, and the complications from smallpox vaccination. Henderson rose rapidly, becoming Chief of the EIS program and the Surveillance Section. This included planning for responding to any imported smallpox case, testing the jet-injector gun for smallpox vaccinations, and assessing deployment of the new measles vaccine in Francophone West Africa, which had been developed at Merck Vaccines. D. A.’s pivotal role in the 20-country West and Central African Smallpox Eradication–Measles Control Program, supported by the US Agency for International Development and implemented by the CDC, in partnership with the countries led to his assignment to Geneva to head the global program. Of the 10 countries with the highest smallpox incidences in the world in 1967, 7 were in West Africa.

**Photo 2.**
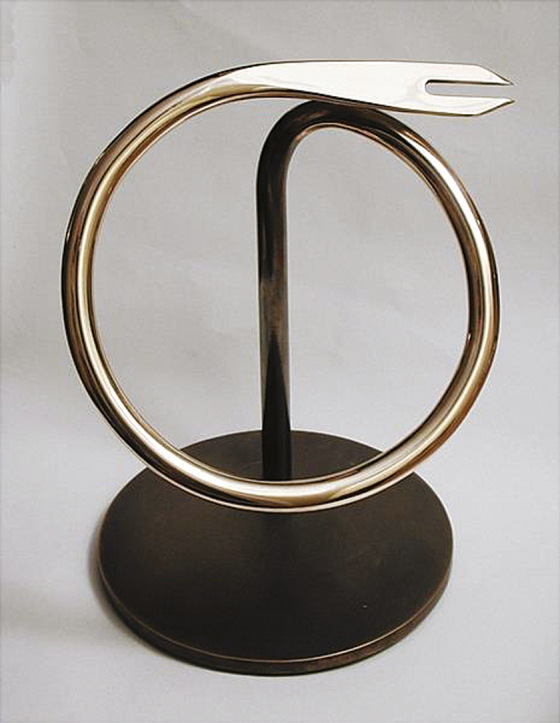
Order of the Bifurcated Needle: 9" bronze model of the lapel pin, made by sculptor David Henderson

The book *Smallpox and Its Eradication*, which D. A. coauthored with others, is an encyclopedic treatise on the history and virology of the disease and a fact-filled chronology of the program [1]. The book does not address the important issue of leadership and D. A.’s essential role in winning the battle against this horrific, ancient scourge. Indeed, D. A.’s qualities were those of the greatest leaders and he applied them advantageously.

**Photo 3.**
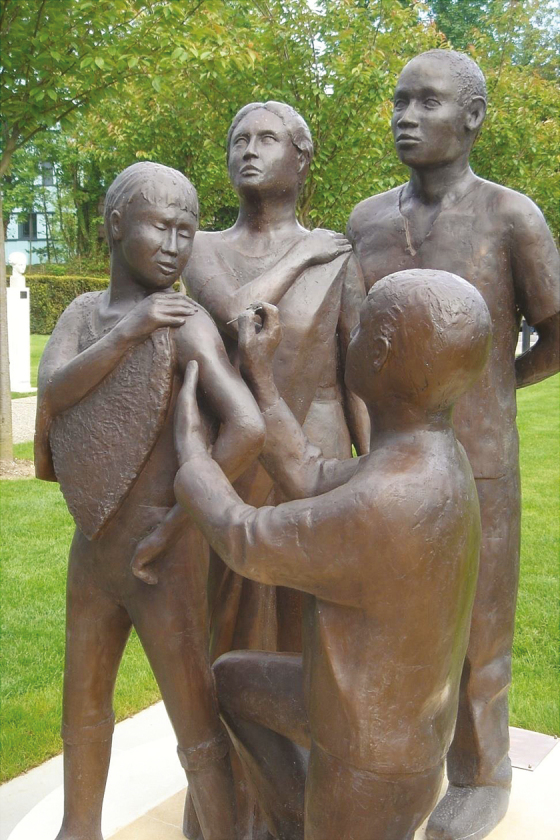
Smallpox Eradication Statue, Unveiling May 17, 2010. Sculptor Martin Williams

• **Communicating the mission often and loudly.** This was “Smallpox Target Zero,” removing the usual focus on numbers of vaccinations performed. This goal was painted on the sides of buildings and printed on T-shirts, bumper stickers, and the cover of WHO magazines. At every smallpox-related meeting and social gathering, attendees raised their glasses, repeating the mantra, “To zero.” D. A. even had his email address as DAHzero@aol.com.
• **Having good judgment, being honest and forthright—**but bending the laws when needed. Henderson referred to one WHO regional office as “unable to function as a good post office.” The requested funds, supplies, and equipment were sent directly to the requesting country program with copies of correspondence paid personally by D. A. The original transaction letters were forwarded to the Regional Office, as required by WHO protocol, where they might be acted upon weeks or months later.
• **Being receptive to contrarian views.** The program began as a mass vaccination campaign in many countries. When it was shown in West Africa by Bill Foege, a CDC-trained missionary living in eastern Nigeria, that the “ring vaccination/surveillance-containment” strategy was vastly better than indiscriminate mass vaccination, D. A. embraced and promoted this strategy. Smallpox eradication was no “veni, vidi, vici” vaccination program as some might think. Although vaccination was crucial, by the late 1960s it was shown that the disease could be rapidly eliminated by active case finding, epidemiologic analysis, and rapid containment of outbreaks through ring vaccination and community mobilization.
• **Being creative in introducing new technology.** The Ped-O-Jet vaccination gun, developed by Aaron Ismach for the US Army, and adapted to use a hydraulic foot pump in place of electricity, was used in Africa and Brazil where large numbers of people could be assembled, in the mid-1960s to early 1970s. This tool was very effective for delivering smallpox and measles vaccines using different nozzles but required spare parts and much maintenance. The bifurcated vaccination needle invented by Benjamin Rubin of Wyeth Laboratories revolutionized smallpox vaccine delivery, decreasing and simplifying the training and sterilization time for vaccination and the amount of vaccine used. Once confirmed as effective, the bifurcated needle replaced all other forms of vaccination against smallpox in the 1970s. Only heat-stable, freeze-dried smallpox vaccine was used during the program. Production in selected countries adhered to exacting standards established by a WHO advisory group and quality-monitored by Connaught Laboratories in Canada and the National Institute of Public Health in the Netherlands.
• **Promoting partnerships and choosing good lieutenants.** Foremost were the outstanding national leaders such as Bangoura Alécaut in Guinea, M. I. D. Sharma in India, Ato Yemane in Ethiopia, Abdoulaye Deria in Somalia, and Petrus Koswara in Indonesia. D. A. did not choose those, but he, John (Jock) Copland, John Wickett and the administrators in Geneva supported them. Tata Industries in India, the Swedish International Development Agency, US Peace Corps, and Japanese Peace Corps volunteers were a few of the important partners. Key players were the >700 lieutenants (>150 from the CDC) from 73 countries serving in the field from 3 months to several years; all had to become independent and creative managers. Everyone became an international civil servant. This was before computers, cellphones, faxes, social media, geographic information systems, and remote sensing were available. An example was the all-star epidemiologist Ciro de Quadros. He served several years in Brazil’s eradication program. D. A. brought him to Ethiopia for another 6 years. There was no effective road system, so travel by foot with or without a mule was routine for weeks or months at a time. De Quadros documented 26000 cases when the country was reporting a few hundred annually. Another was Nicole Grasset; she was the smallpox program regional coordinator in the WHO Southeast Asian Regional Office who developed a conduit directly to Prime Minister Indira Gandhi. D. A. found Nicole doing relief work in Biafra, eastern Nigeria, in the late 1960s.
• **Promoting allegiance from field staff.** D. A. managed by example, from the front. He and others in the WHO 4-room, maximum 10-person nerve center were constantly in the field, learning new approaches, solving problems, courting donors, transferring successful ideas, and twisting arms. D. A. knew how to motivate others. He said the best ideas for conquering smallpox came from the field—and not from headquarters staff. These included house-to-house searches, use of smallpox identification cards, school searches, monetary rewards for finding smallpox cases, feeding the patients in their homes, use of the famous “Imprest Account” (advance petty cash funds), screening for “fever-and-rash” illness at major pilgrimages and festivals, and the ring vaccination-case containment strategy.
• **Being decisive and optimistic.** D. A. was outwardly confident about smallpox eradication despite political upheaval, civil wars, kidnapped helicopters and teams, natural disasters, staff burnout, and discovery of a new disease, human monkeypox, which resembled smallpox. He was decisive and would follow through, addressing each challenge systematically, while delegating responsibility for finding solutions. D. A. was detail oriented and followed through, but he was not a micromanager.
• **Providing incentives and rewards.** No one became rich working on smallpox. As it became clear by 1976 that Target Zero was getting close. D. A. conceived a special award and supported its manufacture and distribution; his daughter, Leigh Henderson, twisted over 700 bifurcated needles into handsome lapel pins—one of the most coveted awards in medicine, the Order of the Bifurcated Needle **(see photo 2).**
• **Managing and communicating, the keys to eradication.** The in-house rule at the smallpox program headquarters was that requests and letters must be answered within 2 days. Urgent requests, often for vaccine, vehicles, staff, or unraveling political and administrative snafus had top priority. Reports and manuscripts were edited personally by D. A. and sent back to authors within 2 weeks. D. A. started a series of smallpox eradication reports at headquarters. During his 10 years as smallpox unit chief, he read, edited, and distributed to the field >230 reports, most from field staff, many of whom were not lyrical in English. After much effort, D. A. finally got the WHO *Weekly Epidemiological Record* to publish clear and concise country-by-country updates on smallpox incidence, special problems and solutions: fieldworkers could see how they were doing compared with other states and countries (a strong motivator) and share information.
• **Knowing the importance of socializing and humor.** D. A. was tall, robust, and would command an audience with his deep stentorian voice—whether it be with ministers or village watchmen. He and his wife Nana welcomed scores of pox fighters and friends to their homes in Geneva and Baltimore. This proved particularly useful when Viktor Zhdanov, the Russian representative to the World Health Assembly, after appreciating a grilled steak at the Hendersons, invited D. A. to come to Moscow and discuss poor vaccine quality and staff recruitment—during the Cold War.

D. A. went into public health because he was attracted to having communities, countries, and continents as his patients. His house calls were to villages, smallpox wards, prime ministers, and presidents. The field staff did make house calls—more than one billion of them in India alone.

The legacy of D. A.’s work in the smallpox eradication program is 4-fold—apart from the tens of millions of persons who are living and others without disfiguring scars from smallpox:

1. The WHO Expanded Programme on Immunization and, from this, the current polio eradication program.
2. Credibility of national health ministries, international organizations, and partnerships focused on Target Zero. After the confirmation of smallpox eradication, expanded coalitions began forming with ambitious goals and increased financing to address multiple public health problems regionally and globally. There was and is conviction that the impossible was now possible.
3. Pride of tens of thousands of public health workers—ordinary people who accomplished an extraordinary feat and became extraordinary along the way.
4. The boom in interest in global health as a career for thousands of young idealists who will take on the next seemingly impossible public health challenge and paint a bull’s eye on its back. Many of these persons will be inspired by the statue in front of the WHO building **(see photo 3)** dedicated to the eradication of smallpox or by reading D. A.’s book *Smallpox: The Death of a Disease* to get the real story on how it was done [2].

## Notes

This essay derives from Dr Breman’s comments given at the Memorial Celebration of D. A. Henderson’s life at the Bloomberg School of Public Health, Johns Hopkins University, on November 4, 2016. Other talks were given on Henderson’s career as dean of the School of Public Health at Johns Hopkins, director of the Office of Public Health Preparedness at the US Department of Health and Human Services, and the founding director of the Center for Health Security at Johns Hopkins, now at the University of Pittsburgh Medical Center. Leigh, David, and Douglas Henderson spoke about their father, and video interviews of D. A. addressing his views on smallpox eradication were shown. http://www.jhsph.edu/about/history/in-memoriam/donald-a-henderson/celebration-event-details.html
